# *In Vitro* and *In Vivo* Evaluation of Microparticulate Drug Delivery Systems Composed of Macromolecular Prodrugs

**DOI:** 10.3390/molecules13092136

**Published:** 2008-09-10

**Authors:** Hiraku Onishi, Yoshiharu Machida

**Affiliations:** Department of Drug Delivery Research, Hoshi University, 2-4-41, Ebara, Shinagawa-ku, Tokyo 142-8501, Japan

**Keywords:** Macromolecular prodrug, microparticles, nanoparticles, controlled release, *in vivo* behavior

## Abstract

Macromolecular prodrugs are very useful systems for achieving controlled drug release and drug targeting. In particular, various macromolecule-antitumor drug conjugates enhance the effectiveness and improve the toxic side effects. Also, polymeric micro- and nanoparticles have been actively examined and their *in vivo* behaviors elucidated, and it has been realized that their particle characteristics are very useful to control drug behavior. Recently, researches based on the combination of the concepts of macromolecular prodrugs and micro- or nanoparticles have been reported, although they are limited. Macromolecular prodrugs enable drugs to be released at a certain controlled release rate based on the features of the macromolecule-drug linkage. Micro- and nanoparticles can control *in vivo* behavior based on their size, surface charge and surface structure. These merits are expected for systems produced by the combination of each concept. In this review, several micro- or nanoparticles composed of macromolecule-drug conjugates are described for their preparation, *in vitro* properties and/or *in vivo* behavior.

## 1. Introduction

A lot of macromolecular prodrugs have been developed using various kinds of polymeric carriers, mainly in the field of cancer chemotherapy [1, 2], and found to be useful to control drug release, modify biodistribution or excretion and achieve drug targeting. Recently, biological data on the toxicity and pharmacokinetic behavior of various macromolecules has been compiled, and consequently very safe macromolecules are utilized as drug carriers without toxicity. Synthetic polymers, such as HPMA [3, 4] and poly-(L-glutamic acid) [5, 6], and natural macromolecules, such as dextran [7, 8] and albumin [9, 10], are often used as a drug carriers for the conjugates of antitumor agents. These macromolecules are water-soluble, and their conjugates are also water-soluble; therefore, such conjugates have generally been administered intravenously in a solution dosage form. Macromolecules of more than 4 nm hardly undergo glomerular filtration [11, 12]. Neutral and weakly anionic macromolecules are not subject to interactions with biomacromolecules or cells in the body as compared with cationic or strong anionic macromolecules [11, 13]; therefore, neutral and weakly anionic macromolecules of more than 4 nm have been examined as carriers exhibiting a long systemic circulation, and these macromolecules receive an enhanced permeability retention (EPR) effect at inflammatory sites such as solid tumor tissues [14,15,16,17], leading to the localization of a carried drug at diseased sites. In addition to such drug targeting, the drug release properties are important to achieve effectiveness. Stable bonding between the carrier and drug prevent the drug from being released efficiently. Enzymatic or non-enzymatic cleavage of such a bond is needed in order to expect a pharmacological effect. In many cases, enzymatic hydrolysis and pH-dependent hydrolysis have been utilized to achieve drug regeneration from carrier-drug conjugates [4, 18,19,20]. Thus, macromolecular prodrugs have two strong points, drug targeting to the diseased area and controlled drug release.

These concepts of macromolecular prodrugs have been utilized for various drugs. In particular, macromolecular prodrugs of antitumor agents have been examined for their *in vitro* and *in vivo* features. Doxorubicin (DOX) is a strong antitumor agent, but shows severe toxic side effects such as cardiac toxicity, which limits its clinical use. Various macromolecular prodrugs of DOX have been developed. Conjugates of DOX with HPMA and carboxymethyl-dextran (CM-Dextran) linked via biodegradable peptide spacers exhibited a high antitumor effect as compared with DOX itself, and improved the toxic side effects [19,20,21,22]. These were achieved by drug targeting based on the EPR effect and adequate drug release patterns at the tumor site. Recently, macromolecular prodrugs of camptothecin (CPT) and paclitaxel (PTX) have been examined extensively because these drugs have drawn attention as new strong antitumor agents [23,24,25,26,27]. Several macromolecular prodrugs of CPT or PTX exhibited very high antitumor effects and improved the toxic side effects. In addition, many drugs, including methotrexate, cytarabine and mitomycin C (MMC), have been reported for their macromolecular prodrugs [28,29,30,31]. For MMC, most macromolecular prodrugs have been formed via an amide bond between the NH of the aziridine ring of MMC and the COOH of carboxy polymers or proteins [18, 32]. The bond is subjected to hydrolysis at basic pH, and the hydrolysis rate rules the release pattern. Water-soluble macromolecular prodrugs of MMC have been developed in an attempt to enhance efficacy and improve toxic side effects. In particular, anionic dextran-MMC conjugates exhibited very good suppression of tumor growth in mice bearing S180 solid tumor, and improved the toxic side effects [11]. Furthermore, *N*-succinyl-chitosan-MMC conjugates showed a much higher lifespan increase in mice bearing M5076 liver metastatic tumor as compared with MMC itself [33]. The improved efficacy and toxic side effects by the conjugates appeared to be caused by their long systemic circulation and high accumulation at the tumor site. The biological features of the conjugates were found to be greatly influenced by molecular weight, charge and lipophilicity of the macromolecules, indicating that selection of macromolecules should be a key point. Recently, the biological features of macro- or nanoparticles have been clarified. The particle size and shape, and surface properties, such as lipophilicity and charge, are importantly related with pharmacodynamic or pharmacokinetic properties [34,35,36,37,38,39,40,41,42]. This means that in vivo behaviors of macromolecular prodrugs can be further controlled by their conversion into micro- or nanoparticulate dosage forms. In this review, the preparation of microparticles with macromolecular prodrugs of MMC and their evaluation are firstly stated, and further the drug release profiles or the influence of the particle size on the biodistribution are described [43].

Poly(D,L-lactic acid) (PLA) and poly(D,L-lactic-co-glycolic acid) (PLGA) micro- or nanoparticles have been utilized for controlled release or drug targeting of various drugs. Although these particles are very useful for prolonged release, their release patterns are not always the most desirable. In such a case, covalent linkage between a drug and PLA or PLGA may be useful. PLGA-drug conjugates and their nanoparticles are described in the second section.

As stated above, the concept of macromolecular prodrugs has been utilized mainly for antitumor drugs, but is sometimes applied for other drugs; for instance, steroidal drugs to modify drug behavior *in vitro* and *in vivo* [44,45,46,47]. Steroidal agents are often used for the treatment of various inflammatory diseases, but sometimes bring about severe toxic side effects involving immunological deterioration [48]. For local treatment, the specific delivery of drugs to diseased sites is highly desirable [49,50,51,52]. The preparation and *in vitro* evaluation of dextran-steroid conjugates were reported [45,46,47]. In these conjugates, the release of steroidal drugs is controlled by cleavage of the carboxy ester bond. Recently, we developed chitosan-prednisolone conjugates (Ch-SP) by amide coupling between chitosan (Ch) and succinyl-prednisolone (SP) [53]. Further, microspheres composed of Ch-SP (Ch-SP-MS) were prepared and utilized as oral dosage forms for the treatment of inflammatory bowel disease. Ch-SP-MS and enteric-coated Ch-SP-MS were described for their useful features [54], which were realized by transition properties in the grastro-intestinal tract based on the particle characteristics and their drug release profiles [55].

## 2. Macromolecular prodrugs of MMC and their microparticulate formulations

### 2.1. Gelatin nano- and microspheres containing dextran-MMC conjugate (D-MMC)

**Figure 1:**
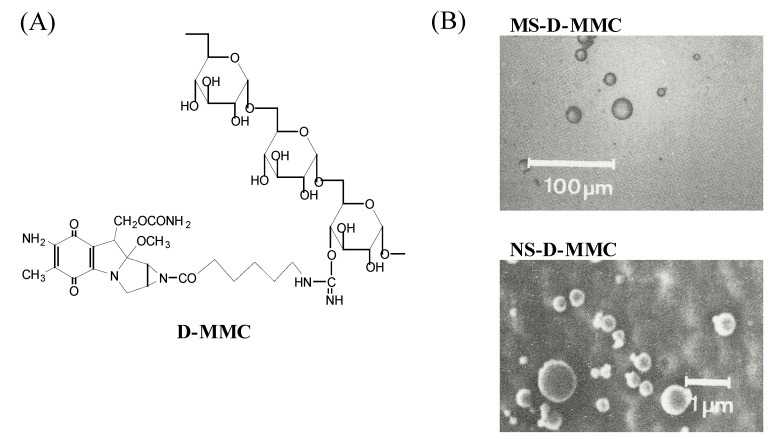
Chemical structure of dextran-MMC conjugate (D-MMC) (A) and scanning electron micrographs of gelatin micro- (MS-D-MMC) and nanospheres (NS-D-MMC) (B).

Dextran-MMC conjugate (D-MMC) has been extensively investigated for its *in vitro* and *in vivo* characteristics (Figure 1). This conjugate is water-soluble, and its physicochemical characteristics, such as charge and molecular weight, can be changed [11,12,13, 56]. As a result, such conjugates exhibit various *in vivo* behaviors, depending on their physicochemical properties. In particular, drug release is clearly controlled by the medium pH [18, 32, 57]. Hydrolytic enzymes barely affect the cleavable bond. Yoshioka *et al*. utilized the release characteristics of D-MMC in the micro- or nanoparticulate systems [43]. Gelatin micro- (MS) and nanospheres (NS) containing MMC and D-MMC were prepared by the W/O emulsification and ice-cooling gelation technique. MS ranged from 5 to 30 μm, and NS was 100 – 600 nm. MMC-containing MS and NS, MS-MMC and NS-MMC, respectively, and D-MMC-loaded MS and NS, MS-D-MMC and NS-D-MMC, respectively (Figure 1), were investigated for their release of MMC in PBS, pH 7.4, at 37^o^C.

MS-MMC released MMC with a 50 % time of 2 h, while NS-MMC released MMC rapidly at the 50 % release time of 10 min, suggesting that particle size markedly influenced the release rate of MMC-containing particles. On the other hand, MS-D-MMC and NS-D-MMC showed almost the same release rate, whereby MMC was released following pseudo-first order kinetics of the 50 % release time of 30 h. Thus, when D-MMC was incorporated, MMC release from particles could be controlled well by cleavage of the amide bond. This was considered due to good retention of dextran with high molelular weight in the particles. Namely, a combination of polymeric microencapsulation and macromolecular prodrugs allowed the release rate to be maintained constantly even when the size of the particles varied.

Biodistribution properties of the particles injected into blood circulation are known to be influenced by their size. In particular, large microparticles (> 7 μm) are subjected to entrapment by the lung, and microparticles of one - several micrometers are easily trapped by the liver and spleen [35]. Hydrophilic nanoparticles of less than a few hundred nanometers show long systemic circulation [39]. NS exhibited high accumulation into the liver, while MS were distributed mainly in the lung. It was considered that MS were trapped by the pulmonary capillary bed due to their size, and NS were phagocytized by the reticuloendothelial system (RES) of the liver and spleen. Such biodistribution profiles of NS and MS resulted in the delivery of MMC specifically into the liver and lung, respectively.

### 2.2. N-Succinyl-chitosan-MMC and 6-O-carboxymethylchitin-MMC conjugate microparicles

**Figure 2:**
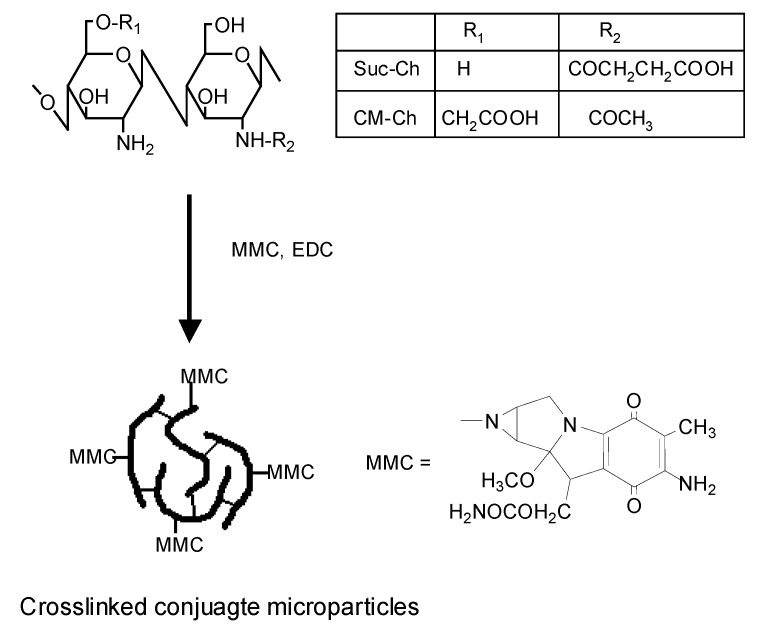
Preparation and structural features of *N*-succinyl-chitosan-MMC conjugate microparticles (Suc-Ch-MMC) and 6-*O*-carboxymethylchitin-MMC conjugate microparticles (CM-Ch-MMC).

MMC can be combined with carboxy groups by amide coupling with carbodiimide. As *N*-succinyl-chitosan (Suc-Ch) and 6-*O*-carboxymethylchitin (CM-Ch) themselves have many carboxy groups, they were considered to be useful for conjugation with MMC [58, 59]. Suc-Ch-MMC conjugate (Suc-Ch-MMC) and CM-Ch-MMC conjugtate (CM-Ch-MMC) were prepared using water-soluble carbodiimide in an aqueous solution. They were obtained as water-insoluble products due to the crosslinking reaction between carboxy groups and remaining amino groups in polymers (Figure 2).

High drug contents could be obtained. The drug release profiles followed almost pseudo-first-order kinetics, but were quite different between Suc-Ch-MMC and CM-Ch-MMC; that is, the 50 % release time was approximately 180 h and 6 h for Suc-Ch-MMC and CM-Ch-MMC, respectively [58]. As Suc-Ch and CM-Ch are very safe polymers, they were considered to be administered parenterally. These solid conjugates could be made into fine particles using a glass homogenizer with a Teflon pestle. The resultant Suc-Ch-MMC and CM-Ch-MMC particles showed an irregular shape near spheres or ellipsoids, and their mean sizes were 4.1 μm (range: 1 – 9 μm), and 7.1 μm (range: 1 – 15 μm), respectively [60]. The tissue concentration of MMC after i.v. injection was investigated. For CM-Ch-MMC, MMC was hardly detected in the blood circulation. At the initial time, CM-Ch-MMC was distributed in the lung to a large extent, and accumulated gradually in the liver and kidney until 5 h after injection. As CM-Ch-MMC was considerably large, it was considered to be entrapped by the pulmonary capillary of the lung. CM-Ch-MMC localized in the lung was considered to release MMC fairly fast, and its elimination from the lung was presumed to be promoted due to the enzymatic hydrolysis of the carrier CM-Ch by enzymes such as lysozyme in the blood and tissues. The gradual accumulation of the drug into the liver and kidney was considered to be derived from small particles generated by degradation of the original particles and released MMC. Suc-Ch-MMC showed similar biodistribution profiles to CM-Ch-MMC, but the elimination rate from the tissue was slower than that of CM-Ch-MMC. A large amount of Suc-Ch-MMC was distributed initially in the lung, probably due to the size characteristics. More than half was maintained in the lung after 5 h, while CM-Ch-MMC was eliminated from the lung to a level of approximately 1/10. This was probably because Suc-Ch-MMC released MMC slower and was hardly biodegraded. The amount of Suc-Ch-MMC accumulated in the liver tended to be more than CM-Ch-MMC. Smaller particles, probably generated by effects such as mechanical breakdown or escape from entrapment by the pulmonary capillary, were presumed to be distributed in RES in the liver. The release of MMC from Suc-Ch-MMC distributed in the lung should also contribute to the increased concentration in the liver and kidney. The histological features of the lung, liver and kidney after i.v. injection of these conjugate particles showed that they did not exhibit significant abnormalities in those tissues for one month. This suggested that the carriers, Suc-Ch and CM-Ch, would be biocompatible. These conjugate particles enabled gradual drug release based on hydrolysis of the amide bond, and the tissue-specific delivery of MMC based on biodistribution features dependent on particle size.

### 2.3. N-Succinyl-chitosan-MMC and 6-O-carboxymethylchitin-MMC conjugate nanoparticles

**Figure 3:**
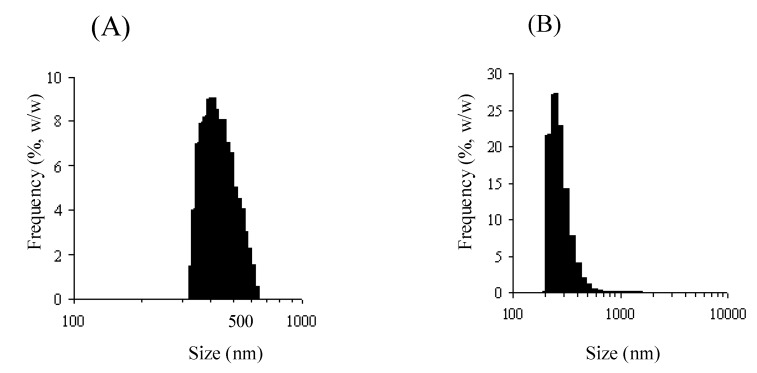
Size distribution of nanoparticles of Suc-Ch-MMC-NP (A) and CM-Ch-MMC-NP2 (B).

As stated above, *N*-succinyl-chitosan-MMC (Suc-Ch-MMC) and 6-*O*-carboxymethylchitin-MMC conjugate (CM-Ch-MMC) formed a solid product by polymer crosslinking. Homogenization of their simple aqueous suspension gave microparticles of several micrometers; however, other techniques were considered to be necessary in order to control their size and shape. In particular, it was quite interesting to produce their nanoparticles, because they might possess the potential to accumulate at inflammatory sites such as tumor, with a so-called enhanced permeability and retention (EPR) effect. Therefore, emulsification and crosslinking in the aqueous phase were applied to prepare nanoparticles of Suc-Ch-MMC and CM-Ch-MMC. As for Suc-Ch-MMC, its nanoparticles (Suc-Ch-MMC-NP) could be prepared by W/O emulsification by sonication (20 min; 28 Hz, 100W) and amide coupling with 1-ethyl-3-(3-dimethylaminopropyl)carbodiimide hydrochloride (EDC). Suc-Ch-MMC-NP had a mean size of 1 mm (range: 230 – 2660 nm) and 450 nm (range: 320 – 660 nm) in the two different conditions (Figure 3) [61].

Although a similar method was applied to CM-Ch-MMC, its nanoparticles could not be produced, and the mean size was more than 10 μm. The droplet size of the aqueous phase in the W/O mixture was considered to influence the size of the particles. Smaller droplets are suitable for the preparation of smaller particles. As it is difficult to obtain fine aqueous droplets from viscous solution, the polymer with a low molecular weight might be better to produce smaller aqueous droplets. Actually, when a CM-Ch with a low molecular weight, named CM-Ch(L0), was used to prepare its particles in a similar manner, nanoparticles (CM-Ch(L0)-MMC-NP) could be obtained and had a mean size of 970 nm (range: 690 – 1420 nm); however, this size was not appropriate to achieve the EPR effect.

The molecular weight and degree of deacetylation of CM-Ch and preparation method of the particles were examined in detail to prepare CM-Ch-MMC nanoparticles (CM-Ch-MMC-NP). CM-Chs of various molecular weights and different degrees of deacetylation were prepared using strong alkaline solution and strong acidic solution. In the novel preparation method, sonication time was prolonged from 20 min to 50 min. When a CM-Ch with a molecular weight of 76,000 and degree of deacetylation of 67 % named CM-Ch(L1) was treated by W/O emulsification and crosslinking with EDC, the obtained nanoparticles exhibited a mean particle size of 370 nm, which was not optimal but was effective for the EPR effect [62]. The conjugate (CM-Ch(L2)-MMC), prepared by the EDC coupling of MMC and CM-Ch with a molecular weight of 51,000 and degree of deacetylation of 83 %, named CM-Ch(L2)), was water-soluble probably due to the low degree of deacetylation. Nanoparticles containing CM-Ch-MMC were prepared by W/O emulsification and crosslinking of CM-Ch(L1) and CM-Ch(L2)-MMC. In crosslinking, CM-Ch(L1) and CM-Ch(L2)-MMC were used at a ratio of 1:5 (w/w). The resultant nanoparticles (CM-Ch-MMC-NP2) showed a mean size of 350 nm (range: 180 – 1220 nm) (Figure 3). CM-Ch-MMC-NP2 exhibited a fairly fast but gradual release of MMC. The detailed properties, in particular, in vivo features, should be further examined in the future.

## 3. Conjugates of drugs with poly(D,L-lactic-co-glycolic acid) and their micro- and nanoparticulate dosage forms

### 3.1. Poly(D,L-lactic-co-glycolic acid) (PLGA)-drug conjugate microspheres

**Figure 4:**
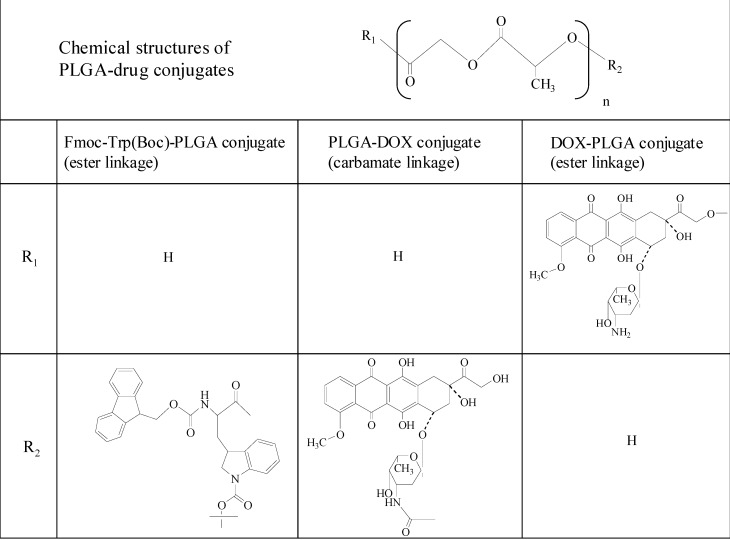
Chemical structure features of PLGA-drug conjugates.

Poly(D,L-lactic-co-glycolic acid) (PLGA) is a polymeric carrier frequently used for the encapsulation of various drugs or proteins because of its high biocompatibility and biodegradability [63,64,65,66]. PLGA micro- and nanoparticles have been utilized as a parenterally injectable dosage form. For such fine particles, it is generally difficult to predictably control the drug release. The release mechanism from PLGA particles includes diffusion in the polymer matrix and erosion of the matrix. In particular, hydrophilic drug-loaded PLGA particles, porous morphology or distribution near the surface brings about initial burst. Release patterns of hydrophilic drugs contained in PLGA particles involve complex diffusion and polymer erosion. In order to avoid these drawbacks of PLGA fine particles, conjugation of PLGA and drugs via a cleavable bond such as an ester was suggested. The release rate depends on the polymer erosion rate and cleavage of PLGA-drug linkage; therefore, PLGA oligomer chains conjugated to the drug and authentic drug are contained as released compounds. Oh *et al*. chemically conjugated poly(D,L-lactic-co-glycolic acid) (PLGA) to a model drug, *N*-(9-fluorenyl-methoxycarbonyl-*N*-*tert*-butoxycarbonyl-l-tryptophan (Fmoc-Trp(Boc)) via an ester linkage (Figure 4) [67]. The conjugation was conducted using two types of PLGA with 50/50 and 75/25 lactic/glycolic compositions, called PLGA50/50 and PLGA75/25, respectively. Microspheres were prepared by O/W emulsification and solvent evaporation using the Fmoc-Trp(Boc) conjugate (100 mg) and PLGA50/50 (400mg), and control microspheres were produced with PLGA50/50 (490 mg) and Fmoc-Trp(Boc). The conjugates were incorporated at 100 % encapsulation efficiency, but conventional microspheres containing free Fmoc-Trp(Boc) showed 20 % encapsulation efficiency. All microspheres had an average size of 8 – 9 μm. Drug release profiles were examined in 0.033 M PBS (pH 7) at 37 ^o^C. Conventional microspheres exhibited an initial rapid release and their release terminated within 5 d. Conjugate micropsheres released the drug over one month at almost zero-order kinetics. PLA chains degrade chemically in physiological pH, and the resultant PLA oligomers with a molecular weight less than 1,050 – 1,150 are water-soluble [68]. Conventional microspheres containing free Fmoc-Trp(Boc) were suggested to release Fmoc-Trp(Boc) by the diffusion control, while conjugate microspheres were considered to modulate the liberation of Fmoc-Trp(Boc)-PLGA oligomers into the medium by the chemical degradation of PLGA chains. Microspheres containing faster degrading PLGA50/50- Fmoc-Trp(Boc) conjugate showed a faster release rate than those with slower degrading PLGA75/25- Fmoc-Trp(Boc) conjugate. HPLC results for the released compounds from conjugate microspheres demonstrated that the released Fmoc-Trp(Boc)-PLGA oligomers are major compounds rather than authentic Fmoc-Trp(Boc). The released Fmoc-Trp(Boc)-PLGA oligomers were expected to be converted into authentic Fmoc-Trp(Boc) by further incubation. These features of Fmoc-Trp(Boc)-PLGA conjugate microspheres suggested a new strategy to control drug release from PLGA microspheres.

### 3.2. PLGA-doxorubicin conjugate nanoparticles

The method utilizing a PLGA-drug conjugate was applied to doxorubicin (DOX). First, DOX was chemically conjugated to a terminal hydroxyl group of PLGA via a carbamate linkage [69], in which the hydroxyl group of PLGA was activated by p-nitrophenyl chloroformate and reacted with the primary amine of DOX (Figure 4). PLGA nanoparticles were prepared by a spontaneous emulsion-solvent diffusion method for PLGA-DOX conjugate and free DOX. The loading efficiency and DOX content of PLGA-DOX conjugate nanoparticles were 97 % and 3.5 % (w/w), respectively, while those values of free DOX-loaded PLGA nanoparticles were 6.7 % and 0.26 % (w/w), respectively.

PLGA-DOX conjugate nanoparticles and free DOX-loaded PLGA nanoparticles displayed a mean size of 270 nm and 360 nm, respectively. Both types of nanoparticles had a high negative zeta potential. The release profiles of DOX in PBS at 37 ^o^C were quite different between the two types of nanoparticles. The release patterns were basically similar to those in Fmoc-Trp(Boc) microspheres. Free DOX-loaded PLGA nanoparticles exhibited an initial rapid release, and the release terminated at approximately one week. PLGA-DOX conjugate nanoparticles released a mixture of PLGA-DOX oligomers in a zero-order fashion over one month. This was caused by the chemical degradation of PLGA-DOX backbone chains, which was confirmed by HPLC analysis. Furthermore, the released fraction from PLGA-DOX conjugate nanoparticles was investigated for antitumor activity by checking the viability of Hepg2 cells by MTT assay. The cytotoxic effect of the released fraction was slightly lower than that of free DOX, which was presumably due to PLGA oligomers attached to DOX. These suggested that novel PLGA-DOX conjugate nanoparticles could be potentially used for DOX release control and drug targeting based on nanoparticulate features.

**Figure 5:**
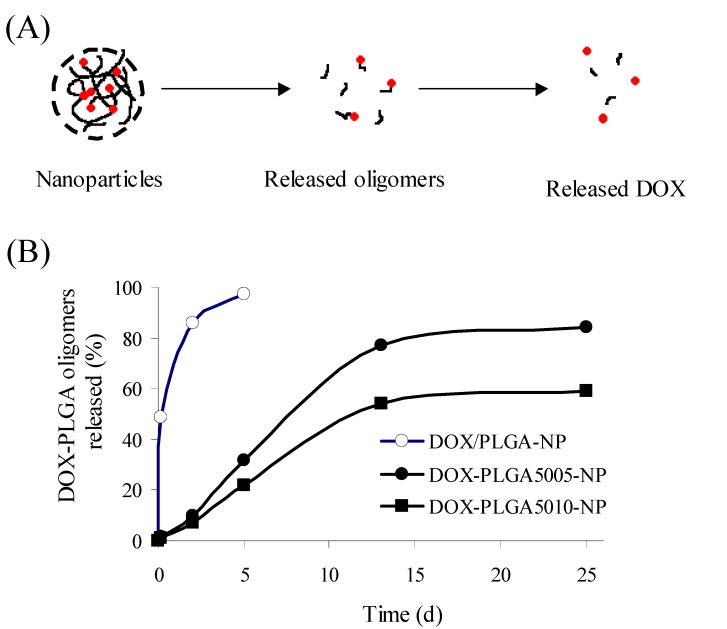
Release scheme of DOX from DOX-PLGA-NP (A) and release profiles of DOX from DOX/PLGA-NP and DOX-PLGA-NP (B).

Another conjugate of DOX with PLGA was prepared by activation of a terminal carboxy group of PLGA, followed by its coupling with a primary hydroxyl group of DOX (Figure 4). In the obtained conjugate (DOX-PLGA), DOX was combined with PLGA by an ester linkage which is expected to be cleavable under physiological conditions [70]. Two types of the conjugate of DOX with PLGA of molecular weights of 5,000 and 10,000, named PLGA5005 and PLGA5010, respectively, were prepared. Nanoparticles using DOX-PLGA conjugate (DOX-PLGA-NP) and a DOX/PLGA mixture (DOX/PLGA-NP) were prepared by solvent diffusion. Both types of nanoparticles were 200 nm. DOX-PLGA-NP exhibited 95 % loading efficiency and 1.9 % (w/w) drug content, while the loading efficiency and drug content of DOX/PLGA-NP were 33 % and 0.67 % (w/w), respectively. DOX/PLGA-NP showed a large initial burst and DOX release duration for another 5 d. DOX-PLGA5005-NP and DOX-PLGA5010-NP displayed more sustained release patterns. DOX-PLGA5010-NP showed a more sustained release pattern than DOX-PLGA5005-NP (Figure 5).

The solubilization of hydrolyzed PLGA fragments takes place when the molecular weight of the fragments becomes around 1,000. Release from DOX-PLGA-NP was considered to be due to the chemical hydrolysis of PLGA chains containing a terminal DOX (Figure 5). The molecular weight influenced that degradation rate. The mode of release was confirmed by HPLC analysis of the released fraction, in which DOX-PLGA oligomers were detected as major compounds. Uptake of DOX-PLGA-NP by Hepg2 cells occurred efficiently, but the cytotoxicity was less than that of free DOX, probably due to the sustained release of DOX-PLGA oligomers or DOX. In an in vivo antitumor test using mice bearing EL4 thymoma subcutaneously, a single administration of DOX-PLGA-NP exhibited a high antitumor effect, comparable to daily-injected free DOX. It was suggested that DOX-PLGA-NP should be useful for highly effective and sustained action of DOX.

## 4. Microspheres composed of chitosan-succinyl-prednisolone conjugate and their enteric-coated microparticles

Recently, in many developed countries, inflammatory bowel disease (IBD), such as ulcerative colitis and Crohn’s disease, has become a critical problem as a severe, chronic and refractory disease as a results of an increasingly westernized lifestyle [71]. IBD is thought to be an inappropriate immune response, and appears to be caused by various factors, including the individual genetic background and environments affecting enteric flora and the intestinal immune system. Steroidal drugs are often used for the treatment of moderate and severe IBD; however, treatment with steroids is often accompanied by toxic side effects, which are mainly based on systemic absorption, that is, non-specific biodistribution. Therefore, therapeutic systems specifically delivering drugs to diseased sites have drawn much attention [72,73,74]. Microparticles, nanoparticles or polymer conjugates containing anti-inflammatory agents have been developed. Microparticles and nanoparticles can control the drug release rate and gastrointestinal transit rate, which are very important for specific delivery to the diseased site. In particular, the transit rate and retention at the diseased site are greatly affected by the particle size. Micro- and nanoparticles with a diameter of less than 10 μm, not subject to elimination by diarrhea, were retained well at the site of colitis with a thicker mucous layer [75, 76]. Small particles can penetrate the mucus layer more deeply. Microparticles of several hundred nanometers to several micrometers are subject to uptake by leukocytes such as macrophages [77, 78], which appear in large numbers at IBD sites. Drug release control is also important in order to suppress drug release before reaching the target sites and achieve subsequent adequate release at the diseased site.

**Figure 6:**
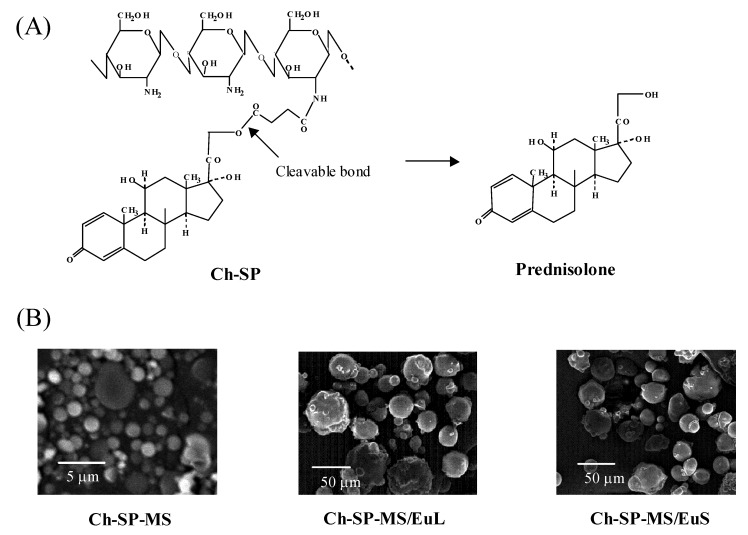
Chemical structure features of chitosan-succinyl-prednisolone conjugate (Ch-SP) (A) and scanning electron micrographs of their microspheres (Ch-SP-MS) and Eudragit L100 and S100-coated Ch-SP-MS (Ch-SP-MS/EuL and Ch-SP-MS/EuS, respectively) (B).

Recently, chitosan (Ch) and its derivatives have been studied for application as drug carriers in oral drug delivery. Ch is mucoadhesive and degraded by intestinal flora. A soluble form has the potential to enhance the permeation of large molecules into the intestinal membrane. A conjugate of succinyl-prednisolone (SP) and Ch, named Ch-SP, was prepared by amide coupling with water-soluble carbodiimide (Figure 6). Acetic acid aqueous solution of Ch-SP was emulsified in liquid paraffin, and the water and acetic acid were evaporated. The resultant microspheres (Ch-SP-MS) (Figure 6) were compared with Ch microspheres physically loaded with prednisolone (PD), named Ch/PD-MS, for particle size, drug content and drug release profiles [53].

Although Ch/PD-MS tended to have a high drug content, the particle size was larger and exhibited an initial rapid release. Ch-SP-MS had slightly less drug content than that of Ch-SP, and a particle size of several micrometers or less. Ch-SP-MS exhibited gradual drug release at neutral and basic pH, but very slow release at acidic pH. Therefore, Ch-SP-MS were superior in release behavior to Ch/PD-MS.

**Figure 7:**
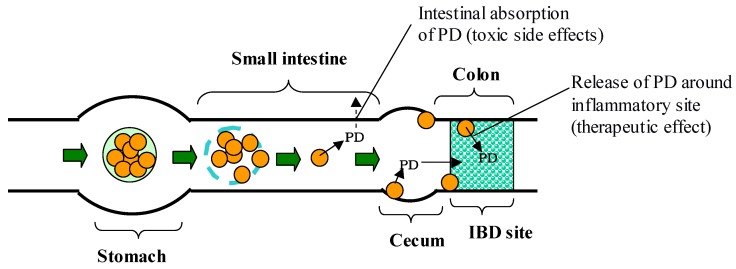
Gastrointestinal transit and PD release after oral administration of Ch-SP-MS/EuL.

Ch-SP-MS swelled extensively and collapsed at stomach pH (pH 1.2). Therefore, they were enteric-coated in order to avoid such swelling or collapse in the stomach [79]. Eudragit L100- and S100-coated Ch-SP-MS, named Ch-SP-MS/EuL and Ch-SP-MS/EuS, respectively (Figure 6), protected Ch-SP-MS from swelling and collapsing at stomach pH, and released Ch-SP-MS at intestinal pH. The release of PD was suppressed at pH 1.2, but increased at pH 6.8 and 7.4. These properties were suitable for the specific delivery of Ch-SP-MS to the lower intestine and specific release of PD. Ch-SP-MS/EuL and Ch-SP-MS/EuS were suggested to be useful as a specific drug delivery system to IBD sites as shown in Figure 7.

Ch-SP-MS/EuL were further evaluated *in vivo* using rats with 2,4,6-trinitrobenzenesulfonic acid (TNBS)-induced colitis [54]. After each rat was fasted for 48 h, TNBS (20 mg) dissolved in of 50 % (v/v) ethanol (0.25 mL) was instilled into the colon in order to induce ulcerative colitis. Three days after TNBS treatment, the substance was administered at 5 mg PD eq./kg via gastric intubation once daily for three consecutive days except for 10 mg PD eq./kg/d, at which administration was conducted twice daily every 12 h for three consecutive days. First, visible damage to the distal colon, stool consistency and rectal bleeding were observed. This visible colitis severity degree (VCSD) was calculated by referring to the report by Tozaki *et al*. [52].

The ratio of proximal colon weight (Cp) to body weight (B), Cp/B, the ratio of distal colon weight (Cd) to B, Cd/B, were calculated to evaluate the inflammation extent. The thymus weight (T) to B, T/B, was determined as an index of toxic side effects. Furthermore, myeloperoxidase (MPO) activity, used as a reliable index of inflammation caused by infiltration of activated neutrophils [76], was measured to quantify the colitis state. Visible colitis severity was suppressed most greatly in the order Ch-SP-MS/EuL > Ch-SP-MS > PD in each score. Cp/B, Cd/B and MPO activity were suppressed most greatly in the order Ch-SP-MS/EuL > Ch-SP-MS > PD, and the suppression was more at 10 mg PD eq./kg than at 5 mg PD eq./kg. PD reduced T/B most greatly, indicating that PD was very toxic. On the other hand, Ch-SP-MS/EuL (10 mg PD eq./kg) displayed almost the same T/B as that of the healthy group, demonstrating that Ch-SP-MS/EuL would be very useful to reduce the toxic side effects of PD. Ch-MS/EuL (carrier alone) showed almost the same values as the control with all assessment parameters. Thus, it was suggested that Ch-SP-MS/EuL should best enhance the efficacy of PD and most reduce the toxic side effect.

Gastrointestinal transit and pharmacokinetics of PD alone and Ch-SP-MS/EuL have been clarified [55]. Namely, after PD alone was administered intragastrically, PD hardly reached the cecum and colon. On the other hand, Ch-SP-MS/EuL delivered Ch-SP-MS efficiently to the lower intestine, and PD was released gradually over 24 h (Figure 7). PD alone gave a high plasma concentration rapidly and was eliminated fast, while the plasma level of PD was suppressed almost completely after administration of Ch-SP-MS/EuL. These *in vivo* results were consistent with the *in vitro* release and regeneration of Ch-SP-MS from Ch-SP-MS/EuL. These pharmacokinetic features supported the superior efficacy and reduced toxicity of Ch-SP-MS/EuL. Thus, enteric-coated Ch-SP-MS, produced by the concept of the combination of a macromolecular prodrug and its microparticulation, were suggested to be useful as an oral delivery system for the treatment of IBD.

In this review, three different types of microparticulate systems, which were composed of or loaded with macromolecule-drug conjugates, were described. Recently, micelle-forming polymeric prodrugs, such as poly(ethylene glycol)-poly(aspartic acid) copolymer-isoniazide conjugate, have been developed [80,81,82,83,84]. These are also produced based on the concepts of passive targeting potential of micelles and controlled release of polymeric prodrugs. Further development and refinement of microparticulate drug delivery systems composed of macromolecular prodrugs will be continued to realize the practical or clinical use.

## 5. Conclusions

Although macromolecular prodrugs have the ability to control drug release and localize at diseased sites, their micro- or nanoparticulation enables further potential to usefully control drug behavior. This is because the biodisposition characteristics of macromolecules and their particulate forms are not the same. As the biodisposition features and gastrointestinal transition profiles of micro- and nanoparticles have been analyzed by various researchers, this knowledge has become available to improve delivery systems. Generally, for micro- and nanoparticles composed of the macromolecular prodrug, drug release is controlled by the prodrug approach, and biodisposition, localization and transit of a drug are controlled by particle characteristics such as size, shape and charge. In this paper, micro- or nanoparticles composed of the macromolecular prodrugs of MMC, DOX and PD were described for their advantageous features and possible usefulness. This concept, combination of a microparticulate system and macromolecular prodrug, is expected to be useful for enhancement of efficacy and improvement of toxic side effect of other drugs. Furthermore, detailed *in vitro* and *in vivo* analyses are critical for practical use or clinical application.

## Abbreviations

Ch: chitosan
Ch-SP: Chitosan-prednisolone conjugate
Ch-SP-MS: Ch-SP microspheres
Ch-SP-MS/EuL: Eudragit L100-coated Ch-SP-MS
Ch-SP-MS/EuS: Eudragit L100-coated Ch-SP-MS
CM-Ch: 6-*O*-Carboxymethylchitin
CM-Dextran: Carboxymethyl-dextran
CM-Ch-MMC: CM-Ch-mitomycin C conjugtate
CM-Ch-MMC-NP: CM-Ch-MMC nanoparticles
CPT: Camptothecin
D-MMC: Dextran-Mitomycin C conjugate
DOX: Doxorubicin
DOX-PLGA-NP: DOX-poly(D,L-lactic-co-glycolic acid) nanoparticles
DOX/PLGA-NP: DOX-poly(D,L-lactic-co-glycolic acid) nanoparticles mixture
EDC: 1-Ethyl-3-(3-dimehylaminopropyl)carbodiimide hydrochloride
EPR: Enhanced permeability retention
Fmoc-Trp(Boc): *N*-(9-Fluorenylmethoxycarbonyl)-*N*-*tert*-butoxycarbonyl-L-tryptophan
IBD: Inflammatory bowel disease
MMC: Mitomycin C
MPO: Myeloperoxidase
MS: Microspheres
MS-D-MMC: D-MMC-loaded microspheres
MS-MMC: MMC-containing micropsheres
NS: Nanospheres
NS-D-MMC: D-MMC-loaded nanospheres
NS-MMC: MMC-containing nanospheres
PTX: Paclitaxel
PLA: Poly(D,L-lactic acid)
PLGA: Poly(D,L-lactic-co-glycolic acid)
RES: Reticuloendothelial system
SP: Succinyl-prednisolone
Suc-Ch: *N*-Succinyl-chitosan
Suc-Ch-MMC: Suc-Ch-MMC conjugate
Suc-Ch-MMC-NP: Suc-Ch-MMC nanoparticles
TNBS: 2,4,6-Trinitro-benzenesulfonic acid
